# A Wearable Iron-Based Implant as an Intramedullary Nail in Tibial Shaft Fracture of Sheep

**DOI:** 10.1155/2019/8798351

**Published:** 2019-03-03

**Authors:** Sitaria F. Siallagan, Marzuki Silalahi, Arief Boediono, Sri Estuningsih, Deni Noviana

**Affiliations:** ^1^Bogor Agricultural University (IPB), Bogor 16680, Indonesia; ^2^Center for Science and Technology of Advanced Materials (BATAN), Tangerang 15314, Indonesia

## Abstract

A stable repaired fracture is the key factor responsible for the recovery of a damaged bone. The iron-based implant is one of the biodegradable metals that have been proven safe as a fracture fixation device. The objective of our experimental approach was to examine the potential of the iron-based implant as a biodegradable metal in tibia shaft fracture in sheep chronically. The samples used for this experiment were iron-based and stainless steel implants. Each had a diameter of 5 mm. These samples were analyzed through 3 phases which are material characterization, in vitro and in vivo examination. The samples were examined using a scanning electron microscope with energy dispersive spectrometer and X-ray diffraction. Based on the analysis carried out, the samples contained 90,02% and 60,81% Fe for iron-based implant and stainless implant, respectively. Both implants maintained high viability when being in contact with calf pulmonary artery endothelial cells, indicating that both implants had a minimum response to the cell in a hemocytometer and methyl tetrazolium (MTT) assay. The systemic effect of the implants was observed using hematology and blood chemistry examination. Data collection also shows that both implants also had a minimum response to the erythrocytes, leucocytes, blood chemistry, and blood mineral during the period of observation. Therefore, it could be concluded that the iron-based implant is tolerable for a period of 9 months. It also has the potential to be used as a biodegradable orthopedic implant.

## 1. Introduction

The main factor that influenced the bone healing is a stable fracture repaired. While the structure of the bone healing is unstable, the intramedullary nail has long been used as a fracture fixation device for tibia shaft fracture. The intramedullary nail is still being used in the veterinary field as needed [1, 2]. The widespread use of stainless steel, titanium, and other nondegradable metal materials is still regarded as the golden standard for the orthopedic implant. This is due to the fact that they are the most applied materials even if more research is still carried out [3, 4].

Nondegradable metals have been used for decades as orthopedic implants for their high strength, ductility, tenacity, hardness, fracture toughness, corrosion resistance, formability, and biocompatibility [5]. Due to the nondegradable metals implant characterizations, a second surgery to remove those implants when the healing process has been completed has proven to be necessary in several cases [6]. Absorbable metals such as magnesium, zinc, and iron are expected to corrode gradually in vivo by an appropriate host response and then dissolve completely upon assisting bone healing [7]. Thus, the requirement of the biodegradable metal implant has increased the research for the iron-based orthopedic implant as a degradable metallic material [8].

Iron is a normal component of heme and nonheme enzymes and proteins [9]. Although the use of iron as a bone implant is still debated due to its toxicity, iron deficiency could lead to a variety of disorder [10]. Iron has attractive mechanical properties, approaching those of stainless steel. Due to the formation of a complex iron phosphate layer, iron has a low degradation rate [11] and is required in little quantity [12]. A lot of iron-based implants research has been conducted, but just a few of them discussed the potential of the iron-based implant in a clinical study chronically.

Numerous orthopedic research has been conducted using sheep as an animal model due to the physical stature and weight [13, 14]. Several orthopedic implants have also been observed in the sheep [15, 16]. However, just a few of these researches observed the iron-based systemic responses as an orthopedic implant. Therefore, the objective of this study is to examine the potential of the iron-based implant as a biodegradable metal in tibia shaft fracture in sheep chronically.

## 2. Materials and Methods

### 2.1. Material Preparation

Iron-based implant (BjPT 6, Tunggal Jaya Steel®, Indonesia) and stainless steel implant (Steinmann Pin, Sklar®, USA) with 5 mm diameter were cut to 1 mm length for material characterization and in vitro examination and were adapted to the tibia bone length size of sheep for in vivo examination. All the implants were sterilized using dry autoclave 121°C for 6 hours and UV ray for 1 hour before being used in this study.

### 2.2. Material Characterization

Surface morphology and metal composition of the implants were observed using a scanning electron microscope (SEM) with an energy dispersive spectrometer (JSM-6510LA, JEOL®) to observe the microstructure of the implants. Iron-based and stainless steel implants were polished using sandpaper until grid # 2000 then etched by using nital (Nital Etch 2%, USA). SEM analysis was taken at an accelerating energy rate of 20 keV. X-ray diffraction with CuK*α* radiation (Empyrean, PANalytical®) was done after the implants were polished until grid #1500 for phase identification

### 2.3. In Vitro Examination

The viability of the cell was examined using hemocytometer and MTT assay. Haemocytometer was used to observe the interference of the implant directly to the cell, and the MTT assay was used to observe the interference of the degradation solution of the implant to the cell. For direct method, the calf pulmonary artery endothelial cell was cultured 10.000 cells per plate in Dulbecco's modified Eagle's medium (DMEM, Sigma-Aldrich) and incubated (Binder, Germany) for 24 hours in 5% CO_2_ and 37°C. Thereafter, an implant with 1 mm of thickness and 5 mm of diameter was put on the surface of the cells and incubated for 76 hours in 5% CO_2_ and 37°C. Half of the media culture was removed, then the rest of it was poured into a 15 ml centrifuge tube (Corning, USA) and centrifuged using flexpin bench-top centrifuge (Tomy, Japan) 500 g for 5 min. The supernatant was removed and the cells were counted using hemocytometer. For the indirect method, all of the implants with 3 mm of thickness and 5 mm of diameter were immersed in low glucose DMEM for 7 and 14 days. One hundred *μ*l of the immersed solution and 100 *μ*l of low glucose DMEM were dripped into 96 plates well (Corning, USA). Ten *μ*l 5 mg/ml MTT stock solution (Sigma Aldrich, USA) was aspirated to each well and then incubated for 4 hours in 37°C and 5% CO_2_ before the supernatant was aspirated. The optical density was read using a microplate reader at 565 nm.

### 2.4. In Vivo Examination

All of the procedures were approved by animal care and use committee of Veterinary Teaching Hospital of Bogor Agricultural University (ACUC RSHP FKH IPB) number 12-2015 RSHP FKH-IPB. Eight male sheep were divided into 2 groups of iron-based and stainless steel implant. Implant with 5 mm of diameter and a length that adapted to the length of tibia bone of the sheep was inserted into the left tibia bone intramedullary.

Before implantation, all of the sheep were injected with an antitetanus serum (Biostat 1.5®, Biopharma, Indonesia). The implants were implanted under general anesthesia. Anesthesia was induced using ketamine and xylazine combination intravenously (3 mg/kg BB Ketamin®, Kepro BV, Netherland and 0.1 mg/kg BB, Xyla®, Interchemie, Holland) and maintained with ketamine intravenously (3 mg/kg BB). Prior to surgery, the surgical site was shaved and iodine solution was applied. A longitudinal skin incision was made to access the lateral diaphysis of the tibia. A middiaphysis tibia osteotomy was performed, stabilizing with the intramedullary bone implant. The wound was closed in three layers, i.e., the fascial, subcutaneous, and skin layers separately. General antibiotics such as gentamycin (3 mg/kg BB, Bio-genta®, Biopharchemie, Vietnam) and amoxicillin long-acting (20 mg/kg BB, Intramox-150 LA®, Interchemie, Holland) were administered intramuscularly to prevent postsurgery secondary infection for 1 week. Tramadol (2 mg/kg BB, Tramadol®, PT Indofarma, Indonesia) was also administered intravenously as an analgesic for 2 weeks. Blood sampling was performed every month until month 9 after implantation on the jugular vein to analyze the systemic effect of the implants.

### 2.5. Data Analytics

Data were analyzed using analysis of variance (ANOVA) and post hoc DUNCAN with the P value of P ≤ 0.05 as statistically significant.

## 3. Results and Discussion

### 3.1. Material Characterization

**Figure 1:**
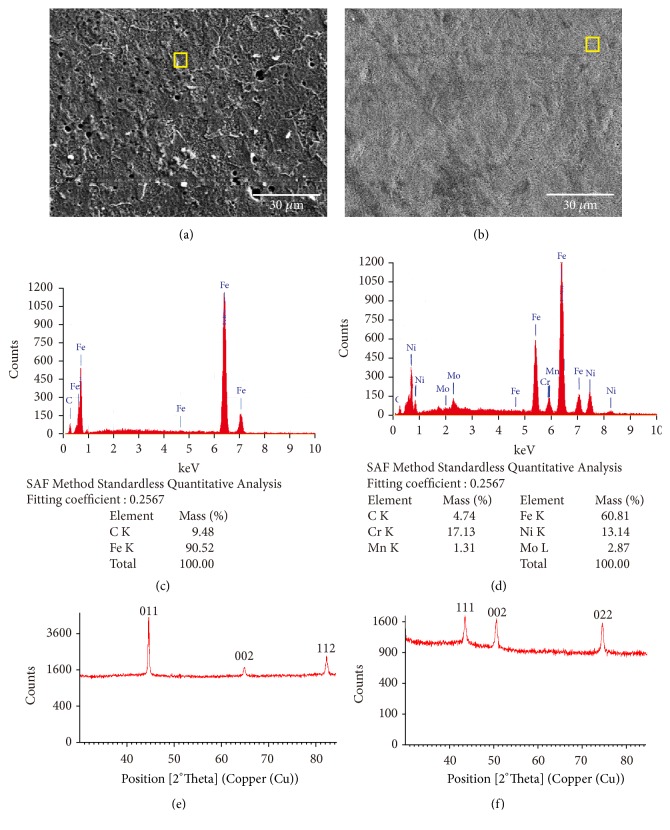
Surface scanning electron microscopy micrographs of sample (a) overview of iron-based implant, (b) overview of stainless steel implant (1000x mag), (c) EDS spectra of iron-based, and (d) EDS spectra of stainless steel, (e) X-ray diffraction of iron-based, and (f) X-ray diffraction of stainless steel implant.

Figure 1 shows the surface morphology of (a) iron-based implant and (b) stainless steel implant. An iron-based implant is seen with small holes and rough surface, while stainless steel implant is well smoothened with smaller holes. According to the SEM and EDS analysis, the iron-based implant contained 90,02% Fe (Figure 1(c)) and the stainless steel implant contained 60,81% Fe; 17,13% Cr; 13,14% Ni; 2,87% Mo, and 1,31% Mn (Figure 1(d)). Figures 1(e) and 1(f) show a plot of observed XRD pattern for sample iron-based and stainless steel implant. The diffraction patterns show the similarity to iron-based steel as indicated by Silalahi et al. [17] and to stainless steel as indicated by Dadfar et al. [18].

### 3.2. In Vitro Examination

**Figure 2:**
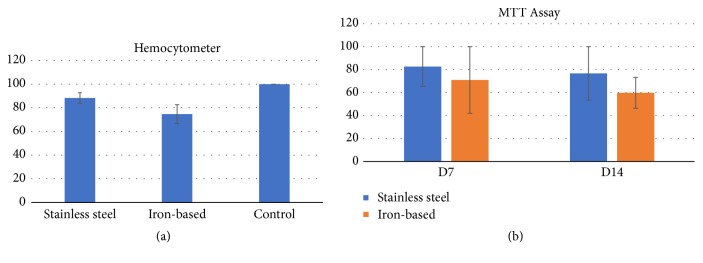
Calf pulmonary artery endothelial cell viability of iron-based and stainless steel implant using (a) haemocytometer and (b) MTT Assay.

Calf pulmonary artery endothelial cell viability of iron-based and stainless steel implant is seen in Figure 2. In hemocytometer examination, both implants had maintained high cell viability of CPAE (Figure 2(a)) during the 3-day incubation time. An insignificant difference (p > 0.05) was observed in both groups, indicating that these implants did not stimulate excessive inhibition in cell culture. Figure 2 also shows that the cell viability of iron-based implant was lower than the cell viability of stainless steel implant using hemocytometer (Figure 2(a)) and MTT assay (Figure 2(b)). Iron is a chemical element that is active in the fundamental physiologic processes [12], but a higher concentration of iron could induce cellular apoptosis [19]. Therefore, the cell motility rate in iron-based implant was higher.

### 3.3. In Vivo Examination

#### 3.3.1. Mineral Concentration

**Figure 3:**
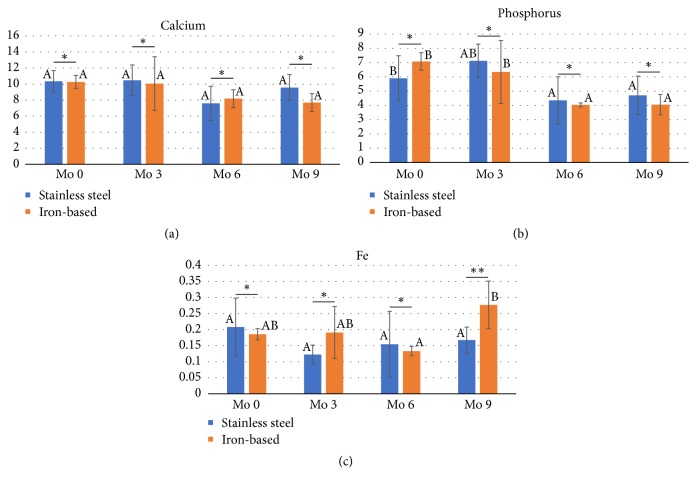
Mineral concentration of iron-based implant in tibial shaft fracture during 9 months' observation time after implantation in sheep (a) calcium, (b) phosphorus, and (c) iron concentration. The same superscript letters (A, B) in same group showed no significant differences during observation time, *∗*insignificant differences and *∗∗*significant difference between both groups in same observation times (P>0.05). Mo: month.

Figure 3 shows the mineral concentration including calcium, phosphorus, and iron of iron-based implant in tibial shaft fracture during 9 months' observation time after implantation in sheep. Insignificant difference in calcium concentration (Figure 3(a)) and significant differences in phosphorus concentration (Figure 3(b)) in each group were seen along with observation time. Iron-based implant shows the calcium concentration slightly decline along the observation time. Fracture healing is characterized by several overlapping stages which result in regenerated bone, namely, an inflammation phase, soft callus phase, and hard callus phase [20]. Calcium and phosphorus are essential bone-forming minerals. These minerals are required for the appropriate osteoblast adhesion, proliferation, and matrix deposition [21, 22]. Calcium is a potential mediator for accelerating the calcification of bone formed [23]; phosphorus plays a critical role in physiological bone matrix mineralization.

Normal iron serum in sheep is varied according to the different authors. The iron serum in this study was still in the normal range [24]. During observation time, the highest iron concentration is at month 9 (Figure 3(c)), indicating iron-ion release in line with observation time [25]. Excess iron facilitates osteoclast differentiation, which is multinucleated giant cells, and bone resorption [26, 27]. Previous studies also have demonstrated that higher iron level will also increase the intracellular ferroportin 1 (FPN-1) in osteoblast and other cells to maintain the iron homeostasis [28]. Iron which is not utilized is stored as ferritin in some cell types. Ferritin stores iron in a nontoxic form and contributes to intracellular iron bioavailability [29].

#### 3.3.2. Erythrocyte Profiles

Erythrocyte profile and thrombocyte of iron-based implant compared to stainless steel implant could be seen in Table 1. Immediately after bone fracture, a hematoma is formed from the bleeding at the fracture site. The hematoma fills the fracture gap in the initial step of bone healing. In the iron-based group, an insignificant decrease of erythrocyte is observed at month 1, in contrast to the stainless steel group. Iron-based implant surface is rougher than the stainless steel surface (Figure 1). Thus, it induced higher hemolysis than stainless steel implant. Bone implant surfaces are meant to positively modulate the interfacial response between the implant and host tissue [30].

At month 4 the number of erythrocytes in the stainless steel group was the lowest from the record gathered and it was significantly different with the iron-based (Table 2). Osteointegration of sheep could be achieved in the 4^th^ month of the healing period [31]. In fracture cases due to hemolysis, lack of erythrocyte will lead to lack of hemoglobin. Bone marrow erythroblasts require a large amount of iron for hemoglobin synthesis [32]. The average lifespan of circulating erythrocytes is approximately 120 days. Within macrophages, hemoglobin derived from phagocytes erythrocytes and free iron is released for circulation [33].

The patterns of erythrocyte, hematocrit, and hemoglobin are alike. An insignificant increase of the patterns indicates light erythropoiesis increases in response to blood loss and inflammation. The increase is caused by the implantation procedures in the early postimplantation, implant-bone tissue interaction, and implant-bone marrow interaction [34, 35].

Thrombocytes, also known as platelets, are the smallest cells of the blood which play a key role in blood clotting and stimulating bone tissue regeneration [36] induced by platelet-derived growth factor [37]. Insignificant changes are also seen in the average number of thrombocyte (Table 1). Although insignificant, the average number of thrombocytes at month 7 is higher than that of month 6 in stainless steel group. The differences are also seen at month 0 and 1. During both observation times, the average number of thrombocytes in stainless steel group is higher than the iron-based group. Thrombocyte increase might be associated with inflammatory, blood loss, or iron deficiency [38].

#### 3.3.3. Leukocyte Count and Differential Leukocytes

**Figure 4:**
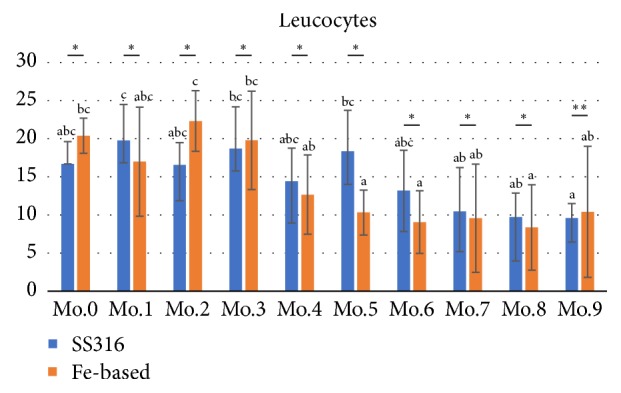
The number of leucocytes in tibial shaft fracture during 9 months of observation time after implantation in sheep. *∗*insignificant differences and *∗∗*significant difference between both groups in same observation times (P>0.05). Mo: month.

Leucocytes, also known as white blood cells, defend the body against disease-causing organisms, toxins, and irritants.  Figure 4 shows the number of leucocytes responses in sheep during 9 months of observation time after iron-based and stainless steel implantation. An inflammatory response could be observed using white blood cell analysis expressed in Table 2. White blood cell examination was performed to analyze the metal hypersensitivity reaction [39] and inflammatory response [40, 41] that could be affected by bone fracture healing [42]. Leucocytes of iron-based implant group show significant changes (Figure 4) during observation time compared to stainless steel implant. Table 2 also shows, almost in all observation times, that the level of leukocyte of iron-based implant is higher than stainless steel implant, especially at month 9.

An inflammatory response of iron-based implant to the body could be observed using leucocyte differentiation analysis which is expressed in Table 2. Eosinophils are a minor circulating granulocyte. They are normally present in low number. Eosinophil-rich inflammation has long been associated with host defense against parasites, promoting allergic reactions [43]. An iron overload could induce eosinophil hyperactivity in mouse and human [44, 45]. In this study, eosinophils data insignificantly changed (Table 2), indicating that the implants have a minimum allergic reaction.

Neutrophils, the first cells recruited to sites of inflammations [46], play a pathos-mechanistic role in compromised bone healing and activated in posttraumatic systemic inflammation [47]. During bone fracture healing, neutrophil synthesizes fibronectin in the gap of fracture [48]. The highest level of neutrophil is seen at month 9 in iron-based implant group (Table 2) along with the highest iron concentration. There is no significant difference in this study, implying minimal inflammation response along the bone healing process.

Bone fracture, as well as the interruption of blood supply and platelet aggregation, induces the release of platelet-derived proinflammatory cytokines, interleukin-6 (IL-6), IL-1, IL-2, and tumor necrosis factor-alpha (TNF-*α*) [49]. These cytokines stimulate the homing of lymphocytes and monocytes/macrophages [50]. In this study, almost in all observation times, the average percentage of lymphocytes of the iron-based group was higher than stainless steel group. Lymphocytes are present at the early inflammatory phase during the initiation of bone repair and in the remodeling phase. The lymphocyte is involved in hard callus remodeling to support osteogenesis [51, 52] with high production of proinflammatory cytokine IL-17 to stimulate the proliferation and osteoblastic differentiation of mesenchymal progenitor cells [53]. Although insignificant, iron-based implant stimulates more severe bone injury than stainless steel implant. This could be caused by the surface of the implant, inducing more soft tissue defect and the concentration of iron that induced the higher inflammatory effect.

Monocyte can differentiate several subtypes of macrophage depending on environmental circumstances. Fractured bone releases the monocyte chemoattractant-1 (MCP-1) [47]. Macrophages play a central role in the organism as they recycle iron after phagocytosis of senescent erythrocytes and also the central contributors to reparative inflammation, coordinating both the injury response and tissue regeneration [53]. Interestingly, the average percentages of lymphocyte and macrophage at months 6 and 7 of the stainless steel group are in contrast to the number of hematocrits. Activated hematocrit attenuates inflammatory response of lymphocyte and macrophage to limit inflammatory processes [54].

#### 3.3.4. Blood Biochemistry of Liver and Kidney

Blood chemistry examination including serum glutamic pyruvic transaminase, serum glutamic oxaloacetic transaminase, blood urea nitrogen, and creatinine could be seen in Table 3. The liver is the primary organ for supporting rapid erythrocyte removal, iron recycling [55], and iron storage [32]. Excess iron would reduce plasma calcium and phosphorus level along with decrease of liver and kidney function [56]. In this study, in line with having no significant differences in the concentration of calcium and phosphorus (Table 3), no significant difference in serum glutamic pyruvic transaminase and serum glutamic oxaloacetic transaminase results indicates the liver function of sheep in both groups is still acceptable.

Majority of metabolic products are excreted via the kidney. Most of the iron in circulation is bound to transferrin, which in the kidney can be taken up via transferrin receptor 1 [57] and reabsorbed by endocytosis in the proximal and distal tubules [58]. Insignificant differences in the iron-based group indicate this implant is still tolerable during 9-month observation time (Table 3). Although the degree of nephrotoxicity is influenced by iron concentration and the duration of iron-nephrocyte interaction [59], the iron level has no significant effects in early chronic kidney disease [60].

Interestingly, there are contradictory results regarding the creatinine index in this study. Creatinine has no useful function and is eliminated by renal glomerular filtration and to a small extent by renal tubular secretion [46]. Creatinine is correlated with skeletal muscle mass. Significant differences are seen during the early three months period in stainless steel group. As mentioned above, hemolysis and lack of erythrocyte will lead to lack of hemoglobin in fracture cases. These conditions will induce kidney to synthesize erythropoietin increase [61], a glycoprotein hormone that regulates erythrocyte production [46], leading to creatinine decrease [62] during early observation time. At month 4, the creatinine level slightly increases probably due to the increased bone density [63]. In human, low creatinine also was positively associated with bone density. The insignificant decrease [64] of creatinine during the last observation time indicates the initial bone density is still not achieved. Generally, this study discussed the biocompatibility effects of the iron-based compared to the stainless steel bone implant in the systemic body of the sheep using blood count, blood mineral concentration, and blood biochemistry examination. To understand more about the local effects of those materials to the bone tissue, histopathology examination should be conducted.

## 4. Conclusions

In this study, the iron-based implant had maintained high cell viability and had induced minimum erythrocyte profile, leucocyte number, leucocyte differential count, and also blood chemistry responses. Thus, it can be concluded that iron-based implant has potential as an intramedullary nail in tibia shaft fracture of sheep.

## Figures and Tables

**Table 1:** Erythrocyte profile and thrombocyte of iron-based implant in tibia shaft fracture in month 0 until month 9 after implantation in sheep.

Mo(s)	Groups	Haemoglobin (g/dL)	Erythrocyte (10^6^/*μ*L)	Haematocrit (%)	Thrombocyte (10^3^/ *μ*L)
0	SS	9.75±0.54^a^	3.43±0.10 ^a^	30±1.41^a^	201.5±91.27 ^a^
	Fe	10.42 ±1.02 ^a^	3.48±0.21 ^a^	30.75±2.63 ^a^	124.25±30.42 ^a^
1	SS	10.95±2.72 ^a^	3.73±0.88 ^a^	33.25±7.93 ^a^	208.75±57.89 ^a^
	Fe	9.53±1.3 ^a^	3.33±0.46 ^a^	29±4.55 ^a^	132.5±35.27 ^a^
2	SS	11±2.27 ^a^	3.63±0.77 ^a^	33.25±6.9 ^a^	151.75±80.85 ^a^
	Fe	11.77±3.04 ^a^	3.98±0.99 ^a^	36±8.91 ^a^	144.25±56.51 ^a^
3	SS	10.47±1.05 ^a^	3.5±0.37 ^a^	31.75±2.99 ^a^	134.75±43.81 ^a^
	Fe	11±0.67 ^a^	3.57±0.29 ^a^	33±2 ^a^	113.5±9,11 ^a^
4	SS	9.53±0.43 ^a, x^	3.13±0.13 ^a, x^	28.5±1.73 ^a^	130.5±61.2 ^a^
	Fe	10.33±0.39 ^a, x^	3.43±0.13 ^a, x^	31±1.41 ^a^	107.75±6.9 ^a^
5	SS	10.55±1.03 ^a^	3.53±0.32 ^a^	32.25±2.63 ^a^	112.75±14.75 ^a^
	Fe	10.76±1.4 ^a^	3.58±0.46 ^a^	32.25±4.11 ^a^	148.25±42.28 ^a^
6	SS	10.98±1.19 ^a^	3.65±0.37 ^a^	33±3.37 ^a^	119.5±15.44 ^a^
	Fe	9.98±0.19 ^a^	3.35±0.1 ^a^	29.75±0.5 ^a^	159.5±42.66 ^a^
7	SS	10.25±0.87 ^a^	3.4±0.29 ^a^	31.25±3.3 ^a^	193±122.52 ^a^
	Fe	11.2±1.22 ^a^	3.7±0.44 ^a^	33.33±3.79 ^a^	134.33±32.56 ^a^
8	SS	10.65±0.83 ^a^	3.53±0.25 ^a^	33±2.45 ^a^	168.5±118.49 ^a^
	Fe	11±0.4 ^a^	3.67±0.15 ^a^	33±1 ^a^	145.67±32.35 ^a^
9	SS	10.58±0.71 ^a^	3.5±0.26 ^a^	31.75±2. 22 ^a^	115.75±6.99 ^a^
	Fe	9.67±1.07 ^a^	3.33±0.31 ^a^	29.66±3.06 ^a^	141.67±44.96 ^a^

Description: data is presented in the average ± standard deviation. The same superscript letters (a,b,c) in different rows but in the same group showed no significant differences (P>0.05). The same superscript letters (x) in different rows and the different group showed no significant differences (P>0.05). Mo: month, SS: stainless steel group, Fe: iron-based group.

**Table 2:** Differential leucocytes of iron-based implant in tibia shaft fracture in month 0 until 9 after implantation in sheep.

Mo	Group	Eosinophil (%)	Neutrophil band (%)	Neutrophil Segmented (%)	Lymphocyte (%)	Monocyte (%)	Basophil (%)
0	SS	0.25±0.5^a^	2±1.41^a^	70.25±8.18^a^	22.5±6.76 ^a^	4±1.82^a^	0±0 ^a^
	Fe	0.75±0.96^a^	1.25±1.5^a^	73±10.13^a^	22.25±10.05 ^a^	2.75±0.96 ^a^	0±0 ^a^
1	SS	1±0.82^a^	2.25±1.71^a^	66±13.37^a^	28±11.23 ^a^	2.75±1.5 ^a^	0±0 ^a^
	Fe	1±0.82^a^	1±1.15 ^a^	62.5±10.21^a^	32.25±8.96 ^a^	3.5±0.58 ^a^	0±0 ^a^
2	SS	0.25±0.5^a^	1.25±1.26^a^	73.25±3.95^a^	21.75±4.35 ^a^	3.5±1.91 ^a^	0±0 ^a^
	Fe	0.5±1^a^	1±1.15^a^	75.75±3.30^a^	20±2.94 ^a^	2.75±2.22 ^a^	0±0 ^a^
3	SS	0.75±0.96^a^	1.75±1.26^a^	71±2^a^	24.5±3.70 ^a^	2.5±1.29 ^a^	0±0 ^a^
	Fe	0.5±1^a^	1±1.15^a^	62.5±9.95^a^	32.75±9.11 ^a^	3.25±2.5 ^a^	0±0 ^a^
4	SS	0±0^a^	0.75±1.5^a^	71.25±5.68^a^	25.5±4.20 ^a^	2.5±1 ^a^	0±0 ^a^
	Fe	0.5±0.58^a^	1.25±1.5^a^	67.75±5.85^a^	28±4.97 ^a^	2.5±0.58 ^a^	0±0 ^a^
5	SS	0.5±1^a^	0.75±0.96^a^	73.25±4.64^a^	18.5±11.39 ^a^	2±2.83 ^a^	0±0 ^a^
	Fe	0.25±0.5^a^	1.75±1.26^a^	69±3.56 ^a^	26±2.94 ^a^	3±1.15 ^a^	0±0 ^a^
6	SS	0.75±0.96^a^	1.25±0.96^a^	63.75±7.41^a^	30±4.97 ^a^	3±0.82 ^a^	0±0 ^a^
	Fe	0.25±0.5^a^	1±1.15^a^	64.25±6.18^a^	34.25±2.36 ^a^	2.75±1.71 ^a^	0±0 ^a^
7	SS	0.75±0.96^a^	1.75±1.26^a^	67.25±12.71^a^	27.75±10.91^a^	2.5±1.29 ^a^	0±0 ^a^
	Fe	1±1^a^	1.33±1.15^a^	67.33±12,34^a^	27±12.12 ^a^	3.33±1.53 ^a^	0±0 ^a^
8	SS	1.5±0.58^a^	2.25±0.5^a^	62±5.48^a,x^	32.5±5.97 ^a, x^	1.75±0.5 ^a^	0±0 ^a^
	Fe	0.67±0.58^a^	2±0^a^	71±1.73^a,x^	23±1 ^a, x^	3.33±1.15 ^a^	0±0 ^a^
9	SS	0.25±0.5^a^	1±1.15^a^	67.75±5.44^a^	28.5±3.87 ^a^	2.5±1.73 ^a^	0±0 ^a^
	Fe	0.67±1.15^a^	2.33±0.58^a^	73±2.65 ^a^	23.67±1.53 ^a^	2.33±0.58 ^a^	0±0 ^a^

Description: data is presented in the average ± standard deviation. The same superscript letters (a,b,c) in different rows but in the same group showed no significant differences (P>0.05). The same superscript letters (x) in different rows and the different group showed no significant differences (P>0.05). Mo: month.

**Table 3:** Blood chemistry results of iron-based implant in tibia shaft fracture in month 0 until 9 after implantation in sheep.

Mo(s)	Groups	SGPT (*μ*/L)	SGOT (*μ*/L)	BUN (mg/dL)	Creatinine (mg/dL)
0	SS	30.25±14.93 ^a^	25±13.44^a^	39.75±2.36 ^d, x^	1.1±0.48 ^c^
	Fe	23.5±12.50 ^a^	31±7.87 ^a^	25±10.39 ^a, x^	0.7±0.22 ^a^
1	SS	27.25±10.78 ^a^	27.25±10.18 ^a^	28.25±5.12 ^abc^	0.95±0.26 ^abc^
	Fe	35±6.21 ^a^	32.75±3.77 ^a^	2925±6.02 ^a^	0.65±0.19 ^a^
2	SS	39.5±5.20 ^a^	28±4.24 ^a^	36±3.65 ^cd^	1.05±0.3 ^bc, x^
	Fe	33±8.49 ^a^	29.75±10.97 ^a^	30±7.26 ^a^	0.45±0.21 ^a, x^
3	SS	26.25±8.5 ^a^	30±12.94 ^a^	23.25±5.67 ^a^	0.53±0.15 ^a^
	Fe	29±9.27 ^a^	36.75±2.87 ^a^	28.75±8.18 ^a^	0.75±0.44 ^a^
4	SS	31.75±12.26 ^a^	25±8.49 ^a^	33,75±4.35 ^bcd^	1.18±0.3 ^d, x^
	Fe	32±6.53 ^a^	27.75±4.86 ^a^	23.5±8.27 ^a^	0.6±0.22 ^a, x^
5	SS	36±6.06 ^a^	28.75±11.41 ^a^	28.5±2.08 ^abc^	0.6±0.18 ^ab^
	Fe	26.5±12.29 ^a^	36.25±10.63 ^a^	27.75±6.55 ^a^	0.6±0.18 ^a^
6	SS	30±16.35 ^a^	30.5±7.14 ^a^	21.75±9.46 ^a^	0.6±0.24 ^ab^
	Fe	33.75±7.27 ^a^	29.25±9.98 ^a^	24.25±4.79 ^a^	0.68±0.22 ^a^
7	SS	29±3.46 ^a^	32.25±3.20 ^a^	25±6.78 ^ab^	0.75±0.33 ^abc^
	Fe	33.67±8.74 ^a^	29±11.53 ^a^	22±8.19 ^a^	0.57±0.15 ^a^
8	SS	25.25±5.32 ^a^	31.25±9.11 ^a^	23±8.04 ^a, x^	0.88±0.40 ^bc^
	Fe	31.67±14.57 ^a^	36.67±11.15 ^a^	36±2 ^a, x^	0.7±0.2 ^a^
9	SS	24.75±6.75 ^a^	30.5±11.79 ^a^	29±3.16 ^abc^	0.6±0.16 ^ab^
	Fe	34±10.58 ^a^	27.67±11.72 ^a^	32±9.16 ^a^	0.87±0.31 ^a^

Description: the data is presented in the average ± standard deviation. The same superscript letters (a,b,c) in different rows but in the same group showed no significant differences (P>0.05). The same superscript letters (x) in different rows and the different group showed no significant differences (P>0.05). Mo: month, SS: stainless steel, Fe: iron-based implant, SGPT: serum glutamic pyruvic transaminase, SGOT: serum glutamic oxaloacetic transaminase, BUN: blood urea nitrogen.

## Data Availability

The data used to support the findings of this study are included within the article.
